# An injury awareness education program on outcomes of juvenile justice offenders in Western Australia: an economic analysis

**DOI:** 10.1186/1472-6963-12-279

**Published:** 2012-08-28

**Authors:** Kwok M Ho, Elizabeth Geelhoed, Monica Gope, Maxine Burrell, Sudhakar Rao

**Affiliations:** 1 University of Western Australia, Perth, Australia; 2 Royal Perth Hospital, Perth, Australia

**Keywords:** Cost benefits, Hospitalization, Injury prevention, Juvenile justice

## Abstract

**Background:**

Injury is a major cause of mortality and morbidity of young people and the cost-effectiveness of many injury prevention programs remains uncertain. This study aimed to analyze the costs and benefits of an injury awareness education program, the P.A.R.T.Y. (Prevent Alcohol and Risk-related Trauma in Youth) program, for juvenile justice offenders in Western Australia.

**Methods:**

Costs and benefits analysis based on effectiveness data from a linked-data cohort study on 225 juvenile justice offenders who were referred to the education program and 3434 who were not referred to the program between 2006 and 2011.

**Results:**

During the study period, there were 8869 hospitalizations and 113 deaths due to violence or traffic-related injuries among those aged between 14 and 21 in Western Australia. The mean length of hospital stay was 4.6 days, a total of 320 patients (3.6%) needed an intensive care admission with an average length of stay of 6 days. The annual cost saved due to serious injury was $3,765 and the annual net cost of running this program was $33,735. The estimated cost per offence prevented, cost per serious injury avoided, and cost per undiscounted and discounted life year gained were $3,124, $42,169, $8,268 and $17,910, respectively. Increasing the frequency of the program from once per month to once per week would increase its cost-effectiveness substantially.

**Conclusions:**

The P.A.R.T.Y. injury education program involving real-life trauma scenarios was cost-effective in reducing subsequent risk of committing violence or traffic-related offences, injuries, and death for juvenile justice offenders in Western Australia.

## Background

Injury is responsible for two-thirds of deaths in those under the age of 24 in Australia and is also associated with a significant number of hospitalizations, disability and costs [1]. In Western Australia, about 2,500 to 3,000 hospitalizations each year are related to injuries from motor vehicle accidents [2]. The population incidence of head injury, mostly due to motor vehicle accidents or assaults, in Western Australia is approximately 20 per 100,000 person-years (95% confidence interval: 17–22), resulting in severe disability in a significant number of young people [3,4]. In 2010 in New South Wales, young people aged between 15 and 24 years old were involved in 53,863 road traffic crashes that included approximately 97,290 private motor vehicles, resulting in a total of 14,988 fatalities and injuries [5]. In 2010, the estimated total cost incurred by road traffic accidents by young people aged between 15 and 24 years old in New South Wales alone was 1488 million, and in 2003 the total annual cost of motor vehicle accidents in Australia was approximately $17 billion and accounted for about 2.3% of the Gross Domestic Product [5,6].

Many assaults, motor vehicle accidents and physical injury in developed countries are related to risk-taking behaviors involving alcohol and drug use [7,8]. Health promotion strategies including economic and retailer interventions, alcohol taxation, reducing alcohol availability, legal and legislative strategies, and strategies addressing the servers of alcohol have been shown to be effective in reducing driving under the influence of alcohol [9]. Evidence to support the cost-effectiveness and economic aspects of many health education interventions in reducing physical injury however remains uncertain [10,11].

Our recent study showed that participation in an injury education program involving real-life trauma scenarios was associated with a reduced subsequent risk of committing violence or traffic-related offences, injuries, and death for juvenile justice offenders [12]. We hypothesized that from a health service perspective this injury education program is cost-effective, and conducted an economic analysis on this injury awareness program for juvenile justice offenders.

## Methods

### Participants and setting

The P.A.R.T.Y. (Prevent Alcohol and Risk-related Trauma in Youth) program is a one-day youth injury prevention program developed in 1986 by the Regional Trauma Centre at Sunnybrook and Women’s College Health Sciences Centre in Toronto, Canada. The program provides relevant information to young people to improve their awareness of injury-producing situations, make informed prevention-oriented choices, and adopt behaviours and actions to minimize risk of injuries. Royal Perth Hospital was the first hospital in Australia that initiated the P.A.R.T.Y. education program in 2006.

Although the main focus of this program was on high school students (between grade 10 and 12), this program was also provided to traffic-related juvenile justice offenders (aged between 14 and 18 years old) who were referred to this program by juvenile justice court magistrates on an ad hoc basis. Currently this education program takes part once per week for high school students and once per month for juvenile justice offenders. The participants of this program spend a day following the admission of an imaginary major trauma patient. After attending talks on pre-hospital care and the vulnerability of the brain and spinal cord to injuries, participants visit the Emergency Department, Intensive Care Unit (ICU), and Trauma wards. The participants are shown why and when a serious injury is more likely to occur and are given the opportunity of talking to trauma patients about their experiences and attempting to mobilize using a wheelchair and crutches, giving them an insight into previously unconsidered consequences of risk-taking behaviour.

### Effectiveness data

During the study period, the referral of juvenile justic offenders to this education program was on an *ad hoc* basis by the discretion of the court magistrates as part of the juvenile justice offenders’ rehabiliation program. All those who were referred to the program had attended the program. Among a total of 3659 juvenile justice offenders who were sentenced by court magistrates during the study period, 225 were referred to the P.A.R.T.Y. education program. A substantial proportion of the participants of the program (57%) stated that the program would modify their attitude on risk-taking behaviors and also expressed very positive comments about the values of the program [12]. Using data from the WA Department of Health and WA Police, we obtained the health and offence outcome data of all juvenile justic offencers included in this study. The median follow-up period for the whole cohort was 33 months (IQR: 17–44 months).

Those who were referred to the injury awareness program were slightly different from those who were not referred to the program. Court magistrates tended to select more males, subjects without prior offences, and European or Indigenous subjects to attend the P.A.R.T.Y. injury awareness education program (Table1). Age, nature of offences and socioeconomic factors were not different between those who were referred to the program and those who were not.

**Table 1:** Difference in characteristics of juvenile justice offenders who were referred and those who did not get referred to to the injury prevention program

**Variables**	**Referred (n = 225)**	**Not referred (n = 3434)**	**P value**
Age, years (SD, IQR)	16.3 (2.0, 16–17)	16.0 (1.6, 15–17)	0.089
Male, no. (%)	191 (85)	2261 (66)	0.001
Ethnic groups, no. (%)			0.001
**-** Caucasian	103 (46)	1082 (32)	
**-** Indigenous	42 (19)	391 (11)	
**-** Asian	7 (3)	24 (0.7)	
**-** Others	19 (8)	67 (2)	
**-** Unknown	54 (24)	1870 (54)	
Nature of offences leading to 1^st^ referral to juvenille justice system during the study, no. (%):			0.221
- assault-related	55 (24.4)	973 (28.3)	
- traffic-related	170 (75.6)	2461 (71.7)	
Mean Index of Relative Socioeconomic Advantage and Disadvantage (SD, IQR)	1009 (53, 973–1049)	1004 (64, 964–1049)	0.142
Number of prior offences prior to the study (SD, IQR)	0 (0, 0–0)	0.1 (0.5, 0–0)	0.001

^#^ P value was generated by Chi-square or Mann–Whitney test. SD, standard deviation. *IQR* interquartile range.

**Figure 1:**
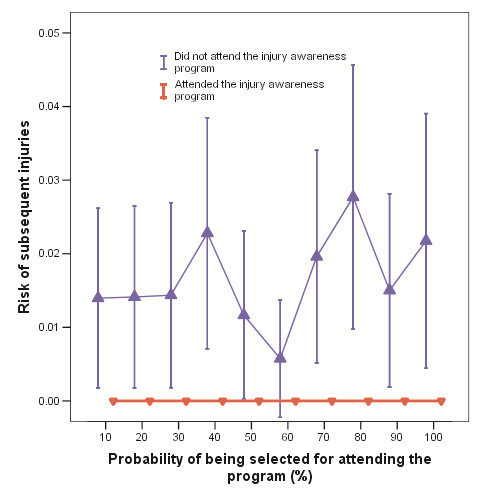
**Interactions between effect of the education program on risk of subsequent injuries and probability of being referred to the education program.**[12].

The incidence of subsequent injuries leading to hospitalization (0% *vs.* 1.6%, including 0.2% fatality; absolute risk reduction [ARR] =1.6%, 95% confidence interval [CI] 1.2%-2.1%; number needed to benefit = 62) was significantly lower for those who had attended the program compared to those who had not [12]. Because selection bias may affect the validity of the effectiveness data, a propensity score based on age, sex, ethnicity, number of offences prior to the study, and socioeconomic background as defined by Index of Relative Socioeconomic Advantage and Disadvantage (IRSD), representing the probability of juvenile justice offenders being selected for the injury education program, was generated for each study subject to assess the potential effect of selection bias. Using a technique similar to assessing calibration of a prognostic model [13,14], we did not observe any interactions between the probability of being selected to attend the education program and the effectiveness of the program in reducing the risk of subsequent injuries (Figure1).

Apart from reducing risk of subsequent injuries, this education program was also effective in reducing traffic or violence-related offences (3.6% *vs.* 26.8%; ARR 23.2%, 95%CI 19.9%-25.8%; number needed to benefit = 4.3, 95%CI 3.9-5.1; p = 0.001), as were alcohol or drug-related offences (0% *vs.* 2.4%; ARR 2.4%, 95%CI 1.9%-2.9%) [12].

### Economic analysis

In this economic analysis, the following assumptions were adopted in the base model.

1. According to the Australian Institute of Health and Welfare, the average cost of hospitalization in a public hospital in Australia was $4,706 in 2009–2010 (http://www.aihw.gov.au/hospital-performance-cost/). We validated this information using average length of stay of the study subjects who had been hospitalized for injury during the study period and mutiplied this by the reported average cost of one day stay in an acute hospital to estimate the acute hospital cost per injury for the study subjects [15].

2. The program required employment of one full-time senior trauma nurse and some consumables for the participants. The total costs of running this education program was about $150,000 per year and resources allocated to the juvenile justice offenders was about 25% of this program ($37,500 per year). The talks provided by the medical or nursing staff to the participants of this program other than by the designated injury education program trauma nurse were treated as part of their clinical duties and no additional cost was needed.

3. When estimating the cost per life year gained, we assumed the average age of participants as 20 years old and their average life expectancy was 80 years if they survived at the end of the follow-up period of this study.

4. When analysis cost per life year gained, both an undiscounted estimate and a discount rate of 3% in estimiting future benefits gained were presented [16].

5. Juvenile justice offenders included in this study did not emigrate out of Western Australia before the end of study period.

6. This economic analysis was from a health service perspective and costs were referenced to 2010.

### Sensitivity analyses

1. This was a pilot program with only a small number of juvenile justice offenders referred (6%), and it is reasonable to expect that a larger program could be implemented with a small marginal cost for each additional attendee. In the sensitivity analysis, we assumed that we could increase the frequency of the program from monthly (on average 5 juvenile justice attendees per month) to twice per month, three times per month, or weekly (5 juvenile justice attendees per week), that is, an increase in the capacity of the program by a factor of 2, 3 or 4, without a significant increase in the overall costs of the program.

2. We assessed whether changing the discount rate of life year gained from 3% to 2, 4 or 5% will change the results significantly.

3. We assessed the cost benefit of this program by including non-hospital costs of serious injury in the analysis ($22,217 per serious injury) [5].

## Results

During the study period, there were 8869 hospitalizations and 113 deaths due to violence or traffic-related injuries among those aged between 14 and 21 in Western Australia. The mean, median, standard deviation, interquartile range (IQR) and range of their length of hospital stay were 4.6, 1, 12.3, 1–3 and 1–310 days, respectively. A total of 320 patients (3.6%) needed ICU admission with an average length of stay in ICU of 6 days. These data validated that the average cost of hospitalization of this cohort of study subject was very similar to the average cost of each hospitalization reported by the Australian Institute of Health and Welfare.

### Net costs needed and saved

Of those not referred to the program (n = 3434), 56 had subsequent serious injuries leading to hospitalization (1.6%) including five deaths (0.2%) and also 919 recommitted an offence, comprising of 639 traffic offences, 258 assault, and 22 others. Therefore we would expect, based on the same proportions above for those who had attended the injury awareness program (n = 225), four serious injuries leading to hospitalization and 60 re-offenders, comprising of 42 traffic offences, 17 assaults and 1 other. Hence the program has prevented *per year* 0.8 serious injuries resulting in hospitalization, 0.068 deaths and 10.8 offences during the study period of 4 years and 10 months. The annual cost saved due to serious injury requiring hospitalization was $3765 (= $4706 average cost per hospitalization x 0.8) and hence the net cost of running this program for juvenile justice offenders was $33,735 (= annual cost of running the education program for juvenile justice offenders: $37,500 - cost reduction in reducing injuries by the program: $3765).

### Cost-effectiveness analysis

Based on outcomes and costs described above, cost per serious injury avoided was $42,169 (= net cost of running the program: $33,735/injuries prevented per year: 0.8), cost per undiscounted and discounted life year gained were $8,268 (= net cost of running the program per year: $33,735/[number of fatality prevented (0.068) x average life-years lost per fatality (60 life-years)] and $17,910, respectively. The estimated cost per offence prevented was $3,124 (= net cost of running the program per year: $33,735/number of offences reduced per year: 10.8).

### Sensitivity analysis

By increasing the frequency of the education program to once per week, the program would be even more cost-effective. We estimated that the cost per offence prevented would be $781 (total 43 offences prevented), the cost per serious injury avoided would be $10,542 (3.2 major injuries prevented), and cost per life year gained discounted at 3% would be $4478.

**Figure 2:**
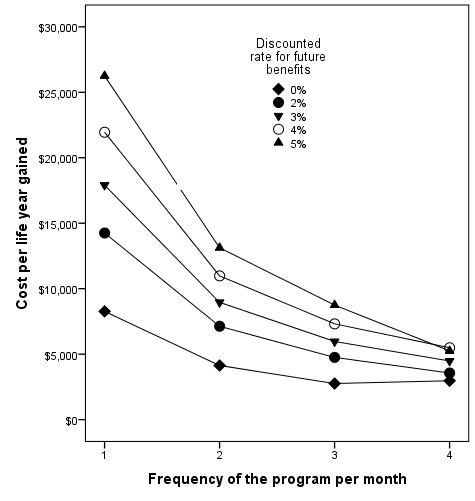
Changes in cost per life year gained of the program by changing the frequency of the education program per month and the discount rate for future benefits.

If we included the post-hospitalization costs of injuries, the net cost of running the program would be $19,726. We estimated that the cost per offence prevented would be $1826, the cost per serious injury avoided would be $24,658, and cost per life year gained discounted at 3% would be $10,472. Even using a higher discounted rate, at 4% or 5%, for future life years gained, the program remained reasonably cost-effective (Figure2).

## Discussion and conclusion

This study showed that the P.A.R.T.Y. injury education program involving real-life trauma scenarios was cost-effective intervention in reducing subsequent risk of committing violence or traffic-related offences, injuries, and death for juvenile justice offenders. This result is significant and requires careful consideration.

First, a recent Access Economic report on economics of road trauma showed that the average cost of a person hospitalized due to motor vehicle accident was much higher than the figure we used in this cost-effectiveness analysis ($22,217 *vs.* $4706) if we included all private and public costs such as rehabilitation, occupational therapy, and home services [5]. Furthemore, the cost of loss in productivity due to injuries and costs related to damage to vehicles and infrastructure, police time, potential legal implications have also not been considered in this economic analysis. As such, if we change our economic analysis from a health service perspective to a societal perspective, the P.A.R.T.Y. education program would be even more cost-effective. Second, the cost-effectiveness of this program compared favorably with some widely accepted preventive programs such as screening for breast cancer - evaluated at $24,000 per life year gained [17] - and also life-saving medical interventions for patients with major trauma [18,19].

Third, this small pilot program is not operating at its capacity and many more juvenile justice offenders can be potentially be accommodated. Currently only two hospitals in Australia, Royal Perth Hospital in Western Australia and Alfred Hospital in Victoria, are offering this education program to juvenile justice offenders. It is thus possible to improve the overall health and health-cost benefit of this program if the program is implemented in other states of Australia and other parts of the world. Fourth, a prospective study on the effectiveness of this education program on a large cohort of high-school students is underway in this study centre. Whether this education program is equally cost-effective for young people without prior offences in Australia remains uncertian. If the program is also proved to be useful in reducing serious injuries among the high school student participants, for example achieving a relative risk reduction of 30%, a reduction in up to 4 and 2 motor vehicle accident deaths per 100,000 population for males and females respectively, 600 hospitalizations, and $510 million health care cost each year in Western Australia alone is possible [2,6,20]. In addition, this education program may potentially change the lives of many young Australians and their families who would otherwise be seriously affected by traffic-related serious injuries.

The final consideration is the limitations of this study. First, although selection bias was not apparent when we analyzed the effectiveness of this education program [12], due to the design of the study, hidden or unmeasured selection biases might have, at least in part, contributed to the substantial beneficial effect of this education program and hence the cost-effectiveness of the program. Second, the follow-up period of this study was relatively short (median 33 months). It is possible that by empowering our participants to protecting themselves through the risky period for life-threatening injuries will alter their attitudes towards risk-taking behaviour for the rest of their lives. Whether the benefits of this education program will last for the life-time of the participants remains, however, uncertain and this will affect the results of the cost per life year gained analysis. Third, costs were likely to be overestimated in relation to outcomes because costs were estimated for the entire period, which did not allow for follow-up in outcomes for attendees who attended the program at a later period of the study. As such, a follow-up study on the same cohort of study subjects in a few years time would be warranted to confirm the long-term health and economic benefits of the program. Finally, there are many approaches to promote injury awareness in reducing risk of injuries but the cost-effectiveness of these programs remains uncertain [9,10]. Whether this P.A.R.T.Y. injury awareness program is more cost-effective than other programs or whether they may have synergistic effect on risk of injuries remains uncertain, but this merits further investigation.

In summary, the P.A.R.T.Y. injury education program involving real-life trauma scenarios was cost-effective in reducing subsequent risk of committing violence or traffic-related offences, injuries, and death for juvenile justice offenders in Western Australia. Our results may be useful for policy decision-makers to decide whether this education program should be widely implemented as part of the rehabiliation program for junvenile justice offenders.

## Ethical approval

This study was approved by the Ethics Committees of Royal Perth Hospital (EC2008/071), Department of Health of Western Australia (2011/2), and Western Australia Police (00000FV001). Informed consent was waived by the ethics committees because of the extreme difficulty in obtaining direct contact with juvenile justice offenders and their families, observational nature of the study and the use of deidentified data.

## Abbreviations

ARR: Absolute risk reduction; CI: Confidence interval; IRSD: Index of Relative Socioeconomic Advantage and Disadvantage; ICU: Intensive Care Unit; IQR: interquartile range; P.A.R.T.Y.: Prevent Alcohol and Risk-related Trauma in Youth.

## Competing interests

The authors declare that they have no competing interests'.

## Authors’ contributions

KMH initiated the study, analyzed the data and drafted the manuscript. EG was responsible for analyzing and interpreting the economic data. MG initiated the idea of the P.A.R.T.Y. education program at Royal Perth Hospital and interpreted the data. MB was responsible for collecting the outcome data and interpreting the data. SR was responsible for initiating the P.A.R.T.Y. education program and interpreting the data. All authors agreed with the final version of the manuscript.

## Pre-publication history

The pre-publication history for this paper can be accessed here:

http://www.biomedcentral.com/1472-6963/12/279/prepub
